# Vancomycin rescues sensitivity to isoniazid in clinical isolates of drug-resistant *Mycobacterium tuberculosis*

**DOI:** 10.1128/spectrum.00173-26

**Published:** 2026-09-10

**Authors:** Sukriti Pal, Shalini Birua, K. S. Suhas, Raghu Nandan, Chandrani Thakur, Kushi Anand, Sarika Jain Agrawal, N. Somashekar, Amit Singh, Nagasuma Chandra

**Affiliations:** 1 Department of Biochemistry, Indian Institute of Science 196384, Bangalore, Karnataka, India; 2 Department of Microbiology and Cell Biology, Indian Institute of Science 200195 , Bangalore, Karnataka, India; 3 Centre for Infectious Disease Research, Indian Institute of Science 624437, Bangalore, Karnataka, India; 4 National Tuberculosis Institute 29150, Bangalore, Karnataka, India; 5 Department of Life Sciences, School of Biological & Forensic Science, Kristu Jayanti (Deemed to be University) 344055 , Bangalore, Karnataka, India; 6 Department of Bioengineering, Indian Institute of Science 29120, Bangalore, Karnataka, India; Institut de Pharmacologie et de Biologie Structurale, Toulouse, France

**Keywords:** strategic antibiotic combination, antibiotic rescue, isoniazid resistance, MDR treatment

## Abstract

Tuberculosis (TB) continues to be a major global health challenge, with a particularly high incidence of antibiotic resistance in high-burden countries. The increasing prevalence of resistance in *Mycobacterium tuberculosis* (*Mtb*) to both first-line and second-line TB drugs calls for novel approaches to address this problem. Tackling antibiotic-resistance at its source, with treatment strategies that can reverse resistance or control its new emergence, would be promising in the long run. Our previous work with lab-evolved isoniazid-resistant *Msmeg* showed that resistant bacteria have a diminished capacity to counter oxidative stress, causing them to be sensitive to several drugs like ebselen, phenylarsine oxide, vancomycin, all of which are known to affect redox homeostasis in mycobacteria. In this work, we report the efficacy of the combination, isoniazid+vancomycin (INH+VAN), on several drug-resistant clinical isolates of *Mtb* with varying resistance profiles, isolated from patient sputum samples. We also show that vancomycin is able to rescue sensitivity to isoniazid in a drug-resistant clinical strain of *Mtb* in a murine infection model. Furthermore, by combining transcriptomic analysis and systems-level modeling, we identify that this efficacy is possibly due to a vulnerability arising from cell wall alterations and stress responses during INH-VAN treatment. Our results suggest that the combination INH+VAN is promising by itself or as part of the standard regimens for treating drug-resistant TB cases, and therefore suitable for a clinical trial in isoniazid-resistant and MDR TB cases.

**IMPORTANCE:**

Tuberculosis (TB) is an infectious disease caused by the bacterium *Mycobacterium tuberculosis* (*Mtb*), which claims millions of lives each year, posing a global health challenge. While there are several antibiotic therapies available, resistance to primary drugs severely hinders successful TB treatment. This calls for novel treatment strategies against drug-resistant TB. We have identified a strategy to reverse resistance by addition of a strategically selected repurposable antibiotic that targets the resistance-generating mechanism. Our study demonstrates that vancomycin, in combination with isoniazid, reverses isoniazid resistance and is effective in killing drug-resistant *Mtb*. Since isoniazid is a primary TB drug, and vancomycin is already in clinical use for the treatment of common bacterial infections, they can easily be introduced as a combination into the pipeline for TB treatment. Our work addresses the urgent need for newer TB treatment strategies and provides data indicating the potential for a clinical trial in MDR TB cases.

## INTRODUCTION

Tuberculosis (TB), an infectious disease caused by *Mycobacterium tuberculosis* (*Mtb*), claiming millions of lives each year and for several decades has continued to be one of the world’s most persistent health challenges. Isoniazid (INH) and Rifampicin (RIF) are the most important first-line agents for the treatment of TB. Isoniazid forms an important part of the 4-drug regimen administered through the Directly Observed Treatment, Short-Course therapy for drug-sensitive TB treatment. The treatment regime has two phases: intensive phase given for 8 weeks and a continuation phase given for 16 weeks. Owing to its potency and high bactericidal effect on actively replicating bacilli, INH is used in both phases. Although the main goal of using a combination of four drugs with different mechanisms of action is to reduce the emergence of drug resistance, unfortunately, cases of drug-resistant TB infections are still on the rise. A large number of TB cases are resistant to either isoniazid, or rifampicin, or both, leading to mono-resistant, MDR, or XDR TB infections. There were an estimated 410,000 incident cases of MDR TB in 2022 (1). Globally, MDR TB caused an estimated 160,000 deaths out of 1.3 million deaths due to TB in 2022 (1). These statistics clearly indicate that there is an urgent need for addressing the problem of drug resistance with the discovery of newer therapies. In this work, we focus on tackling isoniazid resistance and propose a drug combination to rescue isoniazid effectiveness.

INH is a prodrug, requiring activation by the bacterial enzyme catalase-peroxidase, *katG*, to form the active drug, an INH-NAD (nicotinamide adenine dinucleotide) adduct (2). This targets *inhA*, an enzyme critical for mycolic acid synthesis in mycobacteria. The inhibition of mycolic acid synthesis, coupled with the generation of free radical species, can cause widespread damage, affecting nucleic acid, protein, and lipid metabolism, contributing toward bacterial death (2, 3). Several mechanisms are reported for the resistance of *Mtb* to isoniazid. Mutations in *katG*, the enzyme that is responsible for the activation of INH, are frequently identified in resistant isolates of *Mtb* (3). The direct target of INH, *inhA*, is also a major hotspot for resistance-conferring mutations (3). The increase in minimum inhibitory concentration (MIC) of INH due to on-target mutations only partially explains the extent of resistance seen in the clinical samples, suggesting that they are not the sole cause of resistance. Indeed, systemic changes in *Mtb* are described in INH-resistant strains (3, 4). Owing to the indirect and widespread effects of this antibiotic, several mutations located away from the primary drug-binding enzymes have also been associated with resistance, such as *ndh* (NADH dehydrogenase), *kasA* (beta-ketoacyl ACP synthase), *fadE24* (fatty acyl-CoA dehydrogenase) (2, 3). INH-resistant *Mtb* also exhibits expression changes of the enzymes associated with the synthesis of long-chain cell wall lipids. Enzymes, such as *kasA*, NATs (*N*-acetyl transferases), *accD* (acetyl carboxylases), and *fabD* (malonyl-CoA ACP transacylase), have been shown to be important for mycolic acid synthesis and found to be upregulated during INH treatment and in resistant isolates (3). Resistance to INH is also influenced by perturbations in NADH metabolism, which alter cellular NADH/NAD^+^ ratios, since the activation of INH is dependent on the cellular pool of NADH. As a result of this, alterations of cellular redox state and several oxidative stress-responsive genes like *ahpC* have also been associated with INH resistance (3). It is therefore evident that antimicrobial resistance in TB involves multiple cellular adaptations, which work either by themselves or simultaneously in a concerted manner.

Often, such adaptations incur a fitness cost on the bacterial cell, giving rise to collateral sensitivities to other drugs (5). A well-known example of collateral sensitivity is the alteration of cell membrane potential associated with aminoglycoside resistance, which, in turn, causes susceptibility to other antibiotics due to the impairment of PMF-dependent efflux pumps (5). Identification of such emergent vulnerabilities allows for the development of effective drug combinations that hinder the bacterium’s ability to adapt. Leveraging these vulnerabilities, in a previous work, we had identified synergy of INH with three different drugs: ebselen, phenylarsine oxide, and vancomycin, against laboratory-evolved INH-resistant strains of *Msmeg* (6). Vancomycin, being an approved drug for the treatment of MRSA, is a promising candidate to be repurposed for the treatment of drug-resistant tuberculosis. The individual MICs of INH (>64 µg/mL) and VAN (>10 µg/mL) against *Mtb* were drastically reduced in combination (INH: 0.125 µg/mL + VAN: 0.078 µg/mL), which provided preliminary support for the high efficacy of this combination against INH-monoresistant and MDR *Mtb* strains (6).

In this work, with an aim to test the suitability of this combination for conducting clinical trials, we assess its effectiveness, (i) *in vitro*, on microbiological cultures of several drug-resistant clinical isolates of *Mtb* obtained from patient sputum samples, and (ii) in a murine infection model. Furthermore, we construct system models of alterations in *Mtb* exposed to isoniazid and identify crosstalk between cell wall alterations and response to stress, which potentially explains the effectiveness of the INH+VAN combination.

## RESULTS

### INH+VAN shows high efficacy in 16 different DR clinical isolates of *Mtb* derived from patient sputum samples

In order to assess the efficacy of the combination across *Mtb* strains with varying genetic backgrounds and resistance profiles, we tested the INH+VAN combination against 19 different clinical strains of *Mtb* isolated from sputum samples of DR-TB patients. We performed a large-scale drug susceptibility test (DST) on the BD Bactec MGIT 960 system, wherein a bacterial inoculum is allowed to grow in the presence of selected antimicrobials at a specific concentration, and bacterial growth quantified in terms of growth units (GU).

All DSTs were performed with four treatment conditions for each strain: untreated (control), INH mono-treatment, VAN mono-treatment, and INH+VAN combination treatment. *Mtb* strains were selected based on their INH resistance level and resistance profiles with respect to other drugs (Table 1). Growth readings for each *Mtb* strain were taken when the corresponding untreated control culture reached a GU of >400 units.

**TABLE 1:** Strain characteristics and growth units of 16 Mtb isolates in each treatment condition. INH-R denotes isoniazid-resistant.

Strain no.	Phenotype	Mutation	INH (growth units)	VAN (growth units)	INH+VAN		
					Growth units (GU)	% Reduction of GU w.r.t INH	% Reduction of GU w.r.t VAN
1	High INH-R	*katG* (S315T)	1,522	2,847	0	100	100
2	MDR	*rpoB* (S531L), *katG* (S315T)	158	417	0	100	100
3	Low INH-R	*inhA* promoter (C15T)	6,311	1,668	134	97.8	91.9
4	Low INH-R	*inhA* promoter (C15T)	15,430	30	0	100	100
5	Low INH-R	*inhA* promoter (C15T)	7,108	157	55	99.2	64.9
6	High INH-R	*katG* (S315T)	11,494	7,139	670	94.2	90.6
7	Low INH-R	*inhA* promoter (C15T)	4,638	553	88	98.1	84.08
8	MDR	*rpoB* (S531L), *katG* (S315T)	9,123	10,513	17	99.8	99.8
9	High INH-R	*katG* (S315T)	4,375	247	32	99.2	87.04
10	Low INH-R	*inhA* promoter (C15T)	208	3,199	82	60.5	97.4
11	High INH-R	*katG* (S315T)	624	1,950	39	93.7	98.0
12	Low INH-R	*inhA* promoter (C15T)	2,898	1,801	0	100	100
13	Low INH-R	*inhA* promoter (C15T)	15,287	1,004	661	95.7	34.1
14	MDR	*rpoB* (S531L), *katG* (S315T)	161	199	131	18.6	34.1
15	High INH-R	*katG* (S315T)	1,222	299	263	78.4	12.04
16	High INH-R	*katG* (S315T)	4,794	540	305	93.6	43.5
S1	INH-S	–	0	5,242	0	–	–
S2	INH-S	–	0	585	0	–	–

The mutational profiles of the strains are listed in Table 1. Line probe assays were performed to screen for the presence of mutations in *katG*, *inhA*, and *rpoB* genes. The strains either harbored an S315T mutation in *katG*, or C15T (nucleotide change at position −15) in the promoter region of *inhA*, combined with *rpoB* S531L in the case of MDR strains. Additionally, mutations in the *ndh* gene, which encodes NADH dehydrogenase and can elevate the intracellular NADH/NAD^+^ ratio, have been associated with INH resistance (7). To evaluate this possibility, we PCR-amplified and sequenced the *ndh* locus. None of the clinical strains harbored mutations in *ndh*, suggesting that altered NADH/NAD^+^ balance does not contribute to INH resistance in these isolates (Fig. S1).

**Fig 1:**
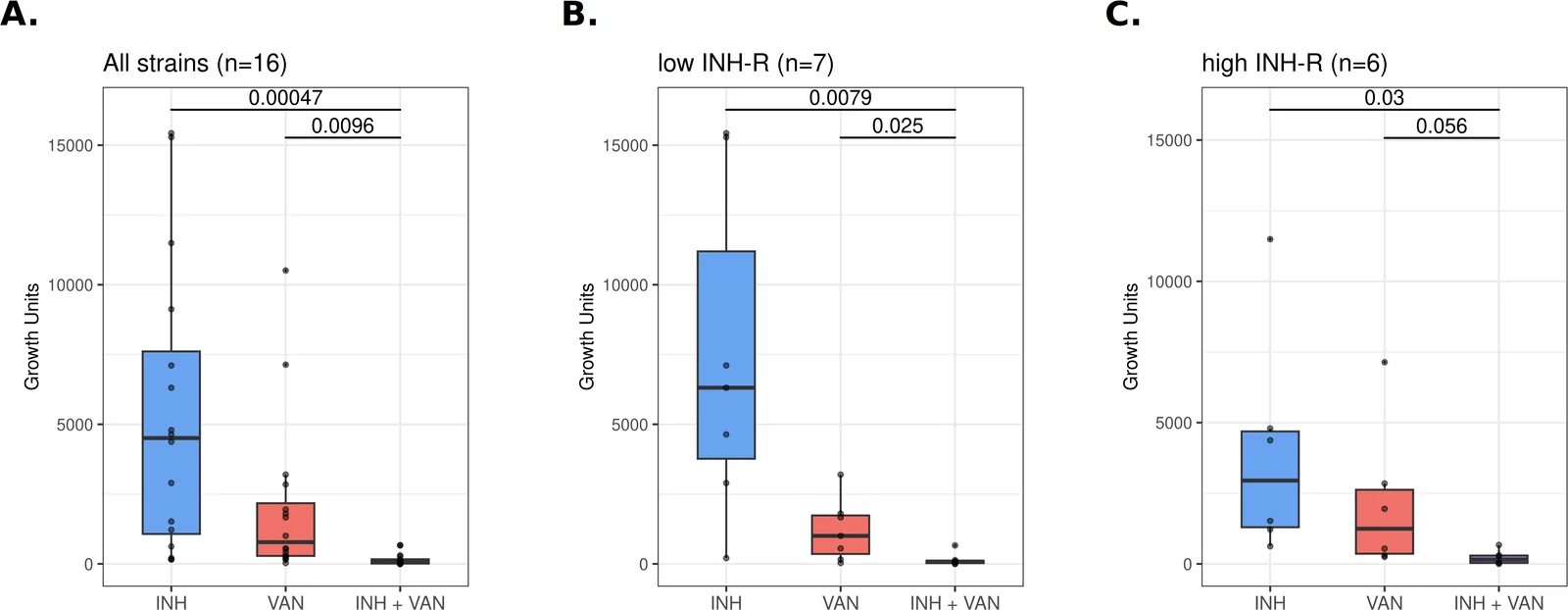
Results of the drug susceptibility tests comparing INH, VAN monotherapy, and INH+VAN combination. (**A**) GU of 16 different clinical isolates of Mtb during different treatment conditions: INH (INH monotherapy), VAN (VAN monotherapy), and INH+VAN (combination treatment). (**B**) Low INH-R group with seven strains, (**C**) high INH-R group with six strains. *P* values reported were obtained from paired *t*-tests. Significant reduction in bacterial growth is observed in the INH+VAN treatment condition as compared to the other treatment groups. Higher growth observed in the “low INH-R” group could be due to lower concentration of INH-treatment as compared to the “high INH-R” group.

Three out of these 19 strains showed no growth in the INH treatment group and were therefore excluded from the study. The remaining 16 *Mtb* strains showed a significant reduction (*P* < 0.01) in growth during treatment with INH+VAN as compared to INH or VAN monotherapy (Fig. 1A). Similar trends are also observed in the low INH-R (*inhA* mutation; INH MIC 0.1–0.2 µg/mL), as well as high INH-R (*katG* mutation; INH MIC ≥ 0.4 µg/mL) groups (Fig. 1B and C). Fourteen out of these 16 strains exhibited >90% reduction of GU in the INH+VAN condition with respect to at least one of the drugs (Table 1), which implies that ~87% of the DR *Mtb* strains in our study were uniquely susceptible to the combination therapy. Taken together, these observations showcase the improved efficacy of INH with the addition of VAN across different clinical *Mtb* strains with varying resistance profiles, indicating a more general effect.

### Effectiveness of the INH+VAN combination in a mouse model of TB infection

**Fig 2:**
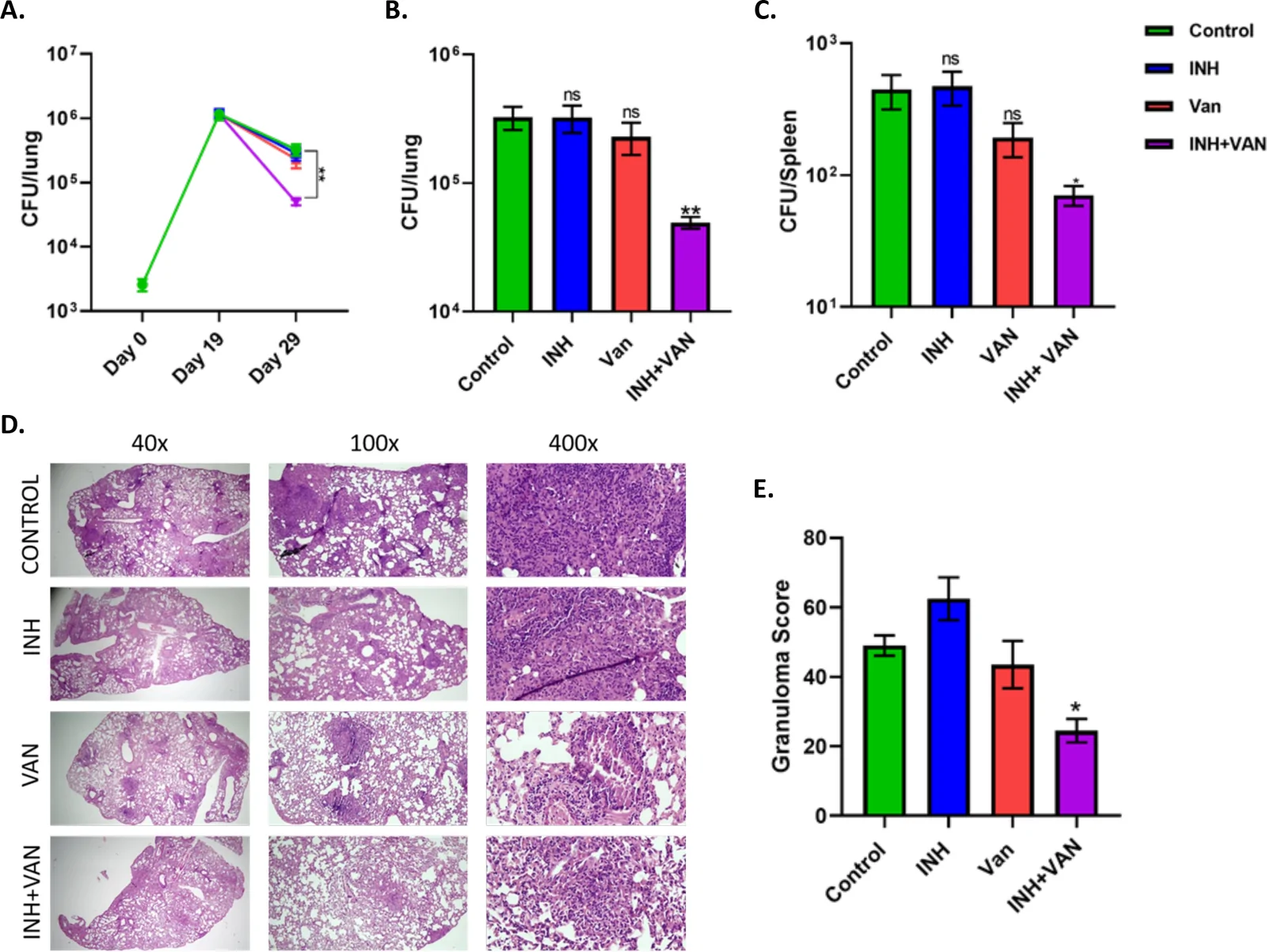
(**A**) Bacterial burden in lungs across time point. (**B**) Day 29 lung CFU. (**C**) Day 29 spleen CFU. (**D**) Representative images of hematoxylin and eosin (H&E)-stained mouse lung sections from control, INH, VAN, and INH+VAN groups. Images in each row are captured at 40×, 100×, and 400× magnifications. Lung sections of infected control and isoniazid treatment groups show greater areas of granulomas with lymphocytic infiltration compared to the vancomycin group. The granulomatous lung lesions are better ameliorated in the isoniazid-vancomycin combination group compared to other groups. Representations of granulomas in each group are depicted in high-power magnification. (**E**) Granuloma score. Statistical significance was determined by one-way analysis of variance with Dunnett multiple comparisons test. The comparison was done against the control (*P = 0.0332, **P = 0.0021, *ns* = not significant). Error bars represent standard deviations from the mean of bacterial burden in 5–7 mice per group.

The therapeutic efficacy of INH, VAN, and their combination against *Mycobacterium tuberculosis* NHN 1664 (MDR strain; BEI resources) was evaluated in an acute model of TB infection in BALB/c mice (Table S1). Bacterial burden was assessed on day 19 post-infection to confirm disease establishment. At this point, lung colony-forming unit (CFU) counts had reached ~10⁶ (Fig. 2A; Table S1), which is notably higher than typically observed for wild-type *M. tuberculosis* H37Rv under similar conditions. This accelerated growth is consistent with the aggressive phenotype of the NHN 1664 strain and the relatively high infection dose (~1,000 CFU at day 0).

Following 10 days of treatment (days 19–29), lung bacterial loads remained high in both the untreated control group and the group treated with INH alone (Fig. 2B), indicating that the NHN 1664 strain is unresponsive to INH monotherapy. Treatment with vancomycin alone showed only a non-significant reduction in bacterial burden. However, the combination of INH and VAN resulted in a significant 10-fold decrease (*P* < 0.05) in pulmonary bacterial load compared to all other groups (Fig. 2B). A similar pattern was observed in the spleen, where neither INH nor VAN alone produced significant effects, but their combination led to the most pronounced reduction in bacterial dissemination of 10-fold (Fig. 2C).

Histopathological examination of lung sections revealed a lesser number of granulomas with or without necrosis in the combination group compared to other groups (Fig. 2D). Accordingly, a significant difference was noticed in the granuloma score of lungs in the combination group compared to the control group, indicating a better clearance of pathological lung lesions (Fig. 2E; Table S2). These observations clearly demonstrate that the addition of VAN renders the MDR *Mtb* strain sensitive to isoniazid *in vivo*, reversing isoniazid resistance.

### Alterations in the cell wall lipid composition upon INH exposure

**Fig 3:**
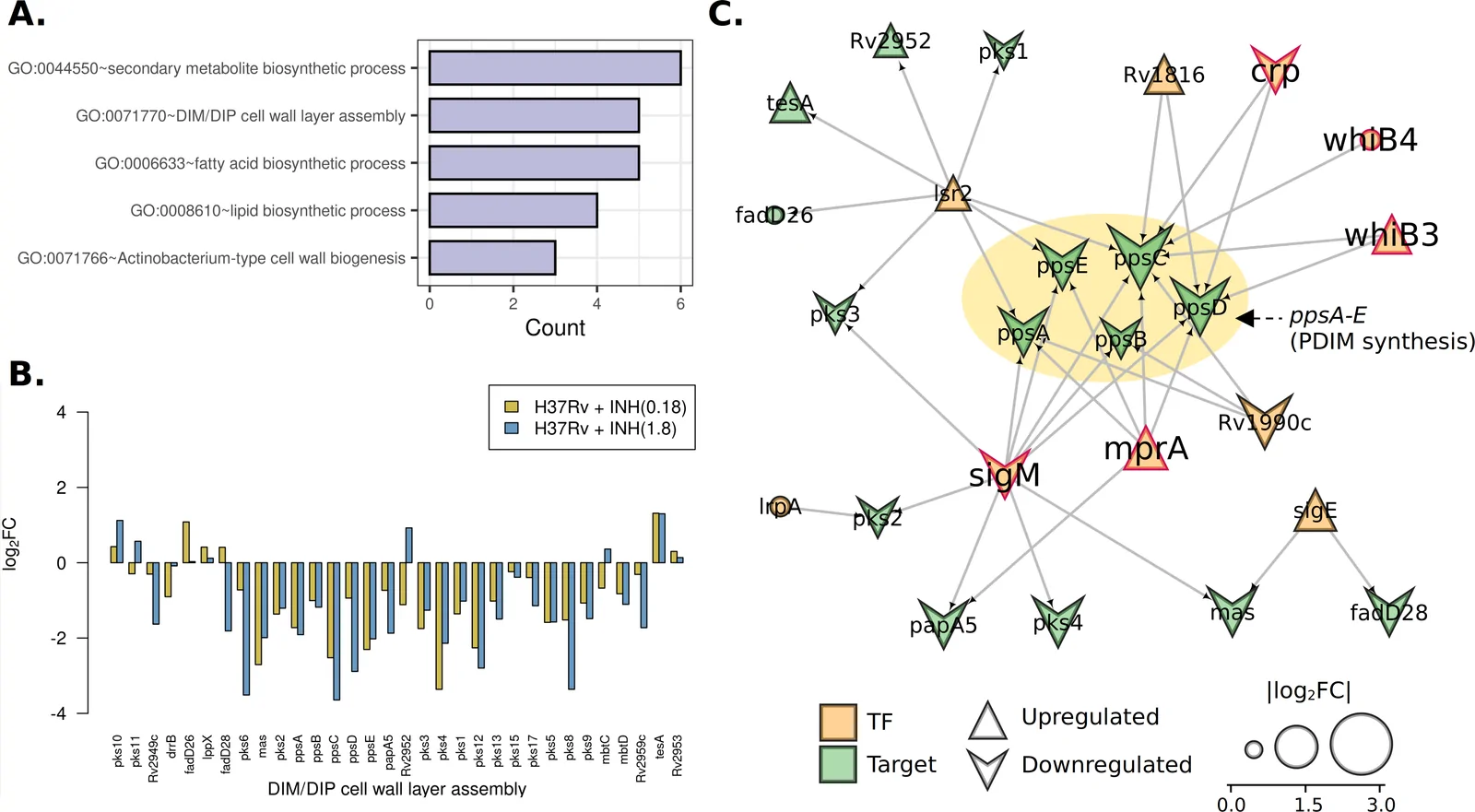
(**A**) Pathway enrichment results for response to isoniazid. Only statistically significant pathways are shown (FDR-adjusted *P* value < 0.05). (**B**) Downregulation of cell wall lipid synthesis during isoniazid treatment (0.18 and 1.8 µg/mL). (**C**) Subnetwork extracted from the transcriptional regulatory network (TRN) topnet of response to isoniazid, showing DIM/DIP lipid synthesis genes along with their regulators. Statistically significant regulators (FDR-adjusted *P* value < 0.05) are highlighted with enlarged text and a red border.

To probe the mechanisms linked to the effectiveness of the combination, we first constructed a network model of protein-protein interactions in *Mtb* and interrogated the alterations that occur upon INH exposure, using publicly available transcriptomic data. Our network is a genome-wide knowledge graph of structural and functional interactions among *Mtb* proteins, contextualized with *Mtb* transcriptomes in different conditions. Using these networks, we applied our in-house developed ResponseNet (8, 9) algorithm to mine top-ranked perturbations that form functional subnetworks and to understand regulatory programs that govern such alterations. We observed that in response to sublethal and lethal isoniazid exposure, several lipid metabolism genes, including those in the mycolic acid pathway, were upregulated, accompanied by a consistent and drastic downregulation of phthiocerol dimycocerosates (PDIM) synthesis genes (*ppsA-E*, log_2_FC ranging from −2 to −4) (Fig. 3A and B). *ppsA-E* encodes a type I modular polyketide synthase associated with the synthesis of phthiocerol and phenolphthiocerol, which form an important part of the lipid and glycolipid layer of the *Mtb* cell wall (10). Downregulation of the *pps* gene cluster indicates alterations of the cell wall lipid composition, which is supported by previous reports showing a lack of PDIM in the cell wall lipidome of *Mtb* treated with isoniazid (11).

To gain systems insights into the molecular events that induce these cell wall lipid alterations, we integrated the transcriptome data with a transcriptional regulatory network (TRN) containing TF-target associations. We then mined this contextualized network to identify the most influential TF-target interactions during isoniazid treatment (Table S3). We further identified statistically significant regulators of cell wall lipid synthesis genes through a hypergeometric test (Materials and Methods). We found five regulators that are most likely associated with the regulation of the cell wall lipid synthesis during isoniazid treatment, of which *whiB3* and *mprA* were upregulated (Fig. 3C). In addition to *whiB3*, *whiB4*, *mprA*, and *lsr2* featured among these significant regulators (Fig. 3C). There is evidence that *whiB3,* in response to redox cues, regulates the production of PDIM and other cell wall lipid components (12). Higher aggregation of PDIM was observed in *whiB3* deletion mutants of *Mtb*, indicating its importance in lipid metabolism (12). Furthermore, there is evidence for the importance of *whiB4* from previously published experimental data, which demonstrate an increase in vancomycin susceptibility during *whiB4* overexpression and decreased susceptibility of *MtbΔwhiB4* mutants to vancomycin (13). WhiB4, an iron-sulfur cluster-containing redox-sensitive transcriptional regulator in *Mtb*, influences cell wall biosynthesis, oxidative stress response, and antibiotic resistance (13–15). The *mprAB* two-component system has been shown to regulate the expression of sigma factors *sigE* and *sigB*, which are known to control cell wall lipid metabolism under stress conditions (16). The direct or indirect regulation of PDIM, mediated via these sigma factors by *mprA*, is further evident from the differential expression of *ppsA-E* in an *mprA* mutant strain of *Mtb* (17). Overall, these regulators respond to multiple stresses like hypoxia, phosphate limitation, and have been shown to play roles during conditions of oxidative stress, virulence, and persistence. In sum, our systems-level analyses clearly indicate that there are significant alterations in the cell wall lipid biosynthesis pathways, especially the downregulation of the PDIMs during INH treatment, providing a possible basis to explain the increased permeability of vancomycin upon exposure to INH, across *Mtb* strains irrespective of their resistance profile.

## DISCUSSION

The growing AMR crisis in TB emphasizes the urgency of developing effective solutions for tackling it, especially to find newer strategies that can potentially curb the evolution and spread of resistance, maximizing the duration of efficacy of antibiotics in clinics. Identification of pivotal resistance mechanisms and targeting them can help in this goal by tackling the problem at its source. Genome-wide alterations in molecular interactions that occur in drug resistance bring out newer dependencies on certain proteins and processes in the resistant bacteria, described as “emergent vulnerabilities,” and the resistant strains are said to be “collaterally sensitive” to compounds that selectively target them (4). In this regard, the use of drug combinations that show patterns of collateral sensitivity in bacteria has high potential, as they hinder the ability of the bacterial cell to adapt when such drugs are used simultaneously. We can therefore leverage these patterns of emergent vulnerabilities associated with AMR for the identification of more effective treatment options that are difficult for the bacterium to overcome. Our previous study with lab-evolved INH-resistant *Msmeg* strains had shown evidence of collateral sensitivity to vancomycin in phenotypic microarray experiments (6).

In this work, we validate the potency of the INH+VAN combination on multiple clinical isolates of *Mtb* with diverse resistance backgrounds, and in mouse models of TB infection, demonstrating that the combination is clearly superior to monotherapy with isoniazid or vancomycin. While vancomycin alone does not exhibit any mycobactericidal effect, it showed high efficacy when coupled with isoniazid in INH-monoresistant and MDR strains.

Intrinsic resistance of *Mtb* to vancomycin is thought to be due to the inaccessibility of its target owing to the encapsulation of the cell wall by a complex lipid layer (10). Our analysis indicates that the cell wall lipid composition of *Mtb* is altered during treatment with isoniazid, including downregulation of PDIM, as a consequence of which vancomycin can access its target when used in combination. This is further corroborated by the action of other cell wall-active antimicrobials against INH-resistant, as well as INH-RIF multidrug-resistant *Msmeg* and *Mtb* strains (6). Consistent with our observations, several studies have reported alterations in the mycobacterial cell wall lipid composition, which cause susceptibility of *Mtb* to vancomycin. *Mtb* isolates deficient in PDIM and/or phenolic glycolipids have been shown to exhibit susceptibility to vancomycin (18–20). Furthermore, mutations that lead to PDIM loss (metabolic deficiency of methylmalonyl-CoA) were shown to increase cell wall permeability, and in turn, vancomycin susceptibility (19). L,D-transpeptidase-deficient *Mtb* are also susceptible to vancomycin and show alterations in cell wall and colony morphology, and aggregation of lipids inside cells, indicating inefficient transport to the cell surface (21). Yet another inhibitor of the mycolic acid pathway (*kasA* inhibitor) is known to be synergistic with vancomycin (22). Increased uptake of vancomycin by mycobacterial cells was observed when they were pre-treated with this inhibitor, hinting at changes in the cell wall lipid composition possibly leading to increased permeability of bacterial cells (22). More generally, vancomycin is seen to be synergistic with long-chain lipid synthesis inhibitors (18).

In conclusion, we show that the INH+VAN combination has proved to be effective *in vivo* against drug-resistant TB isolates, and *in vitro* in several INH-resistant clinical isolates, making it a strong candidate for clinical trials in INH-R and MDR TB. Given that vancomycin is an antibiotic in clinical use for treating other infections, it is more readily available for repurposing for drug-resistant TB. Given the immense need for new alternative treatment regimens for tuberculosis, our study presents the possibility of adding an easily accessible antibacterial to the pipeline for TB treatment.

## MATERIALS AND METHODS

### Assessment of INH+VAN susceptibility of clinical *M. tuberculosis* strains

DNA was extracted from sputum samples using the Genolyse kit (Hain Lifescience GmbH, Nehren, Germany) as per the manufacturer’s instructions. The extracted DNA was processed by Line Probe Assay using GenoTypeMTBDRplus ver 2.0 (Hain Lifescience GmbH, Nehren, Germany) for detection of MTB complex and RIF and/or INH resistance. Drug-resistant clinical strains were selected randomly for inclusion into this study. MGIT 960 vials containing 7 mL Middlebrook 7H9 broth were inoculated, supplemented as described by the manufacturer along with growth supplement (OADC) and PANTA. All inoculated MGIT vials were incubated in the MGIT 960 instrument either until they flagged positive in the instrument or maximum up to 42 days. All positive MGIT vials were confirmed for acid-fast bacilli by ZN staining and further subjected to TB Ag MPT64 Immunochromatographic Assay for identification of *M. tuberculosis* complex. Pure AFB cultures were tested for their sensitivity to INH in MGIT tubes in the BACTEC-MGIT-960 according to the manufacturer’s recommendations (23).

DST was performed at two concentrations of INH: (i) 0.2 µg/mL, and (ii) 0.4 µg/mL. These samples were also tested for susceptibility to VAN at a concentration of 2 µg/mL. The same strains were also subjected to combination DST for INH and VAN at the following concentrations: (i) INH 0.2 µg/mL + VAN 2.0 µg/mL, (ii) INH 0.4 µg/mL + VAN 2.0 µg/mL. Nineteen *Mtb* strains were selected, which were categorized as high-level INH-R (*katG* mutation; INH MIC ≥ 0.4 µg/mL), low-level INH-R (*inhA* mutation; INH MIC 0.1–0.2 µg/mL), or MDR (*rpoB + katG* mutation). H37Rv was used as a drug-susceptible quality control strain. Statistical significance between groups was assessed by performing paired *t*-tests.

### Identification of *ndh* mutations

Genomic DNA was isolated from clinical samples using the GenoLyse kit. Two overlapping fragments of the *ndh* gene were amplified by polymerase chain reaction (PCR) using gene-specific primer pairs (below). The amplified PCR products were purified using either the QIAquick PCR Purification Kit or the QIAquick Gel Extraction Kit (QIAGEN), depending on the specificity of the amplification. Purified PCR products were directly subjected to Sanger sequencing using the corresponding amplification primers:

Ndh2S_F: GCAATGTCCAGGTACTGTTG

Ndh2AS_R: CCTTGGTCGAGTCGATGTG

Ndh3S_F: GACAGATCGCCGAGCTGGC

Ndh3AS_R: TGGACAGGTCGGGCAGCAC

### Assessment of *in vivo* INH+VAN efficacy against MDR *M. tuberculosis*

Female BALB/c mice (6–8 weeks old; *n* = 7 per group) were infected with *Mycobacterium tuberculosis* strain NHN 1664 (~10^3^ CFU/mouse) via the aerosol route using the Glas-Col Inhalation Exposure System. Mice were maintained for 19 days before initiating treatment. Treatment regimens included isoniazid (INH; oral administration, 10 mg/kg), vancomycin (VAN; intraperitoneal injection, 100 mg/kg), or a combination of both drugs, administered daily for 10 consecutive days. An untreated, infected group served as the control.

To confirm infection establishment, a subset of animals was sacrificed on day 19 post-infection, and bacterial burdens were assessed. On day 29 post-infection, all remaining mice were euthanized by isoflurane anesthesia, followed by cervical dislocation. Lungs and spleens were harvested, homogenized in sterile PBS, serially diluted, and plated onto Middlebrook 7H11 agar (BD Difco, Cat. No. 212203) supplemented with OADC enrichment (Cat. No. 211886) and PANTA antibiotic mixture (Cat. No. 245114). Plates were incubated at 37°C for 4 weeks, after which CFUs were enumerated.

Lung sections from each mouse were processed for histopathological examination by hematoxylin and eosin (H&E) staining. Pathological analysis was quantified and represented as granuloma scores.

### Transcriptome analysis and identification of regulators

The RNAseq data set GSE165581 (24) was obtained from NCBI GEO (25), which contains the transcriptional response of *Mtb* H37Rv against lethal and sub-lethal concentrations of isoniazid at different time points. DEG analysis was performed using the *edgeR* (26) and *limma* (27) packages in R. Pathway enrichments of DEGs were carried out using the “enricher” function from the *clusterProfiler* (28) package, using Gene Ontology and KEGG annotations for *Mtb* H37Rv genes in the David database (29).

To identify significant regulators, we used our in-house developed ResponseNet (8, 9) method. Briefly, the method involves contextualizing a TRN with transcriptome data, followed by computation of all-to-all shortest paths and extraction of the top *P* percentile (*P* = 0.3, containing 400 genes, in this study) of the paths ranked by normalized path scores, which capture the most significantly perturbed TF-target interactions. This “topnet,” therefore, provides insights into the regulation of the alterations observed during isoniazid treatment in *Mtb*. For identification of statistically significant regulators in the topnet, we performed a hypergeometric test with the topnet genes belonging to the Gene Ontology pathway, “DIM/DIP cell wall layer assembly,” against each regulator.

Gene log_2_ fold-changes were used to weigh a previously reported *Mtb* TRN (30) as follows:


$$\;\;\;\;\;\;\;\;\;\;\;\;\;\;\;\;NW_{i}=2^{|logFC_{i}\left(\frac{A}{B}\right)|}$$

$$\;\;\;\;\;\;\;\;\;\;\;\;\;EW_{ij}=\;\frac{NW_{i}+NW_{j}}{2\times NW_{i}\times NW_{j}}$$

where *logFC_i_* refers to the log_2_ fold-change of the *i*^th^ gene in condition *A* compared to condition *B*, *NW_i_* refers to the node-weight of the *i*^th^ node, and *A* and *B* correspond to the comparison pair. *EW_ij_* is the edge weight, computed as the inverse of the harmonic mean of *NW_i_* and *NW_j_*. This weighted network was used for the computation of the topnet. Statistical analyses were performed in R v4.4.1, and plots were generated using *ggplot2* (31). Network visualizations were performed on *Cytoscape* v3.10.2 (32).

## Data Availability

All data supporting the findings of this study are available within the paper and its Supplemental Material. RNASeq data analysed in this study are available on the NCBI Gene Expression Omnibus database under the accession number GSE165581 (https://www.ncbi.nlm.nih.gov/geo/query/acc.cgi?acc=GSE165581).

## SUPPLEMENTAL MATERIAL

Figure S1 spectrum.00173-26-s0001.pdf Multiple sequence alignment of the *ndh* locus from *Mtb* H37Rv and 14 clinical isolates presented in this study.

Table S1 spectrum.00173-26-s0002.xlsx Estimation of lung CFUs for the assessment of antibiotic treatment efficacies during TB infection in mice.

Table S2 spectrum.00173-26-s0003.xlsx Histopathological examination and calculation of granuloma scores.

Table S3 spectrum.00173-26-s0004.xlsx Transcriptional regulatory network of top-ranked activities in *Mtb* during INH exposure.

ASM does not own the copyrights to Supplemental Material that may be linked to, or accessed through, an article. The authors have granted ASM a non-exclusive, world-wide license to publish the Supplemental Material files. Please contact the corresponding author directly for reuse.

## References

[1] 2022. Global tuberculosis report 2022. World Health Organization, Geneva.

[2] Timmins GS, Deretic V. 2006. Mechanisms of action of isoniazid. Mol Microbiol 62:1220–1227. doi:10.1111/j.1365-2958.2006.05467.x 17074073

[3] Unissa AN, Subbian S, Hanna LE, Selvakumar N. 2016. Overview on mechanisms of isoniazid action and resistance in *Mycobacterium tuberculosis*. Infect Genet Evol 45:474–492. doi:10.1016/j.meegid.2016.09.004 27612406

[4] Wang X, Jowsey WJ, Cheung C-Y, Smart CJ, Klaus HR, Seeto NE, Waller NJ, Chrisp MT, Peterson AL, Ofori-Anyinam B, Strong E, Nijagal B, West NP, Yang JH, Fineran PC, Cook GM, Jackson SA, McNeil MB. 2024. Whole genome CRISPRi screening identifies druggable vulnerabilities in an isoniazid resistant strain of *Mycobacterium tuberculosis*. Nat Commun 15:9791. doi:10.1038/s41467-024-54072-w PMC11560980 39537607

[5] Pál C, Papp B, Lázár V. 2015. Collateral sensitivity of antibiotic-resistant microbes. Trends Microbiol 23:401–407. doi:10.1016/j.tim.2015.02.009 PMC5958998 25818802

[6] Padiadpu J, Baloni P, Anand K, Munshi M, Thakur C, Mohan A, Singh A, Chandra N. 2016. Identifying and tackling emergent vulnerability in drug-resistant mycobacteria. ACS Infect Dis 2:592–607. doi:10.1021/acsinfecdis.6b00004 27759382

[7] Lee ASG, Teo ASM, Wong S-Y. 2001. Novel mutations in *ndh* in isoniazid-resistant *Mycobacterium tuberculosis* isolates. Antimicrob Agents Chemother 45:2157–2159. doi:10.1128/AAC.45.7.2157-2159.2001 PMC90621 11408244

[8] Ravichandran S, Banerjee U, Dr GD, Kandukuru R, Thakur C, Chakravortty D, Balaji KN, Singh A, Chandra N. 2021. VB_10_, a new blood biomarker for differential diagnosis and recovery monitoring of acute viral and bacterial infections. EBioMedicine 67:103352. doi:10.1016/j.ebiom.2021.103352 PMC8099739 33906069

[9] Sambaturu N, Pusadkar V, Hannenhalli S, Chandra N. 2021. PathExt: a general framework for path-based mining of omics-integrated biological networks. Bioinformatics 37:1254–1262. doi:10.1093/bioinformatics/btaa941 PMC8599850 33305329

[10] Camacho LR, Constant P, Raynaud C, Lanéelle M-A, Triccas JA, Gicquel B, Daffé M, Guilhot C. 2001. Analysis of the phthiocerol dimycocerosate locus of *Mycobacterium tuberculosis*. J Biol Chem 276:19845–19854. doi:10.1074/jbc.M100662200 11279114

[11] Pal R, Hameed S, Sabareesh V, Kumar P, Singh S, Fatima Z. 2018. Investigations into isoniazid treated *Mycobacterium tuberculosis* by electrospray mass spectrometry reveals new insights into its lipid composition. J Pathog 2018:1454316. doi:10.1155/2018/1454316 PMC6029481 30018826

[12] Singh A, Crossman DK, Mai D, Guidry L, Voskuil MI, Renfrow MB, Steyn AJC. 2009. *Mycobacterium tuberculosis* WhiB3 maintains redox homeostasis by regulating virulence lipid anabolism to modulate macrophage response. PLoS Pathog 5:e1000545. doi:10.1371/journal.ppat.1000545 PMC2718811 19680450

[13] Mishra S, Shukla P, Bhaskar A, Anand K, Baloni P, Jha RK, Mohan A, Rajmani RS, Nagaraja V, Chandra N, Singh A. 2017. Efficacy of β-lactam/β-lactamase inhibitor combination is linked to WhiB4-mediated changes in redox physiology of *Mycobacterium tuberculosis*. eLife 6:e25624. doi:10.7554/eLife.25624 PMC5473688 28548640

[14] Chawla M, Parikh P, Saxena A, Munshi M, Mehta M, Mai D, Srivastava AK, Narasimhulu KV, Redding KE, Vashi N, Kumar D, Steyn AJC, Singh A. 2012. *Mycobacterium tuberculosis* WhiB4 regulates oxidative stress response to modulate survival and dissemination *in vivo*. Mol Microbiol 85:1148–1165. doi:10.1111/j.1365-2958.2012.08165.x PMC3438311 22780904

[15] Chawla M, Mishra S, Anand K, Parikh P, Mehta M, Vij M, Verma T, Singh P, Jakkala K, Verma HN, AjitKumar P, Ganguli M, Narain Seshasayee AS, Singh A. 2018. Redox-dependent condensation of the mycobacterial nucleoid by WhiB4. Redox Biol 19:116–133. doi:10.1016/j.redox.2018.08.006 PMC6111044 30149290

[16] Sachdeva P, Misra R, Tyagi AK, Singh Y. 2010. The sigma factors of *Mycobacterium tuberculosis*: regulation of the regulators. FEBS J 277:605–626. doi:10.1111/j.1742-4658.2009.07479.x 19951358

[17] He H, Hovey R, Kane J, Singh V, Zahrt TC. 2006. MprAB is a stress-responsive two-component system that directly regulates expression of sigma factors SigB and SigE in *Mycobacterium tuberculosis*. J Bacteriol 188:2134–2143. doi:10.1128/JB.188.6.2134-2143.2006 PMC1428128 16513743

[18] Soetaert K, Rens C, Wang X-M, De Bruyn J, Lanéelle M-A, Laval F, Lemassu A, Daffé M, Bifani P, Fontaine V, Lefèvre P. 2015. Increased vancomycin susceptibility in mycobacteria: a new approach to identify synergistic activity against multidrug-resistant mycobacteria. Antimicrob Agents Chemother 59:5057–5060. doi:10.1128/AAC.04856-14 PMC4505240 26033733

[19] Mulholland CV, Wiggins TJ, Cui J, Vilchèze C, Rajagopalan S, Shultis MW, Reyes-Fernández EZ, Jacobs WR Jr, Berney M. 2024. Propionate prevents loss of the PDIM virulence lipid in *Mycobacterium tuberculosis*. Nat Microbiol 9:1607–1618. doi:10.1038/s41564-024-01697-8 PMC11253637 38740932

[20] Rens C, Laval F, Daffé M, Denis O, Frita R, Baulard A, Wattiez R, Lefèvre P, Fontaine V. 2016. Effects of lipid-lowering drugs on vancomycin susceptibility of mycobacteria. Antimicrob Agents Chemother 60:6193–6199. doi:10.1128/AAC.00872-16 PMC5038262 27503643

[21] Schoonmaker MK, Bishai WR, Lamichhane G. 2014. Nonclassical transpeptidases of *Mycobacterium tuberculosis* alter cell size, morphology, the cytosolic matrix, protein localization, virulence, and resistance to β-lactams. J Bacteriol 196:1394–1402. doi:10.1128/JB.01396-13 PMC3993333 24464457

[22] Li S, Poulton NC, Chang JS, Azadian ZA, DeJesus MA, Ruecker N, Zimmerman MD, Eckartt KA, Bosch B, Engelhart CA, Sullivan DF, Gengenbacher M, Dartois VA, Schnappinger D, Rock JM. 2022. CRISPRi chemical genetics and comparative genomics identify genes mediating drug potency in *Mycobacterium tuberculosis*. Nat Microbiol 7:766–779. doi:10.1038/s41564-022-01130-y PMC9159947 35637331

[23] Siddiqi SH, Rüsch-Gerdes S. 2006. MGIT procedure manual for BACTEC MGIT 960 TB system. Foundation for Innovative Diagnostics, Geneva, Switzerland.

[24] Srinivas V, Ruiz RA, Pan M, Immanuel SRC, Peterson EJR, Baliga NS. 2021. Transcriptome signature of cell viability predicts drug response and drug interaction in *Mycobacterium tuberculosis*. Cell Rep Methods 1:ne. doi:10.1016/j.crmeth.2021.100123 PMC8688151 34977849

[25] Barrett T, Wilhite SE, Ledoux P, Evangelista C, Kim IF, Tomashevsky M, Marshall KA, Phillippy KH, Sherman PM, Holko M, Yefanov A, Lee H, Zhang N, Robertson CL, Serova N, Davis S, Soboleva A. 2013. NCBI GEO: archive for functional genomics data sets—update. Nucleic Acids Res 41:D991–D995. doi:10.1093/nar/gks1193 PMC3531084 23193258

[26] Chen Y, Chen L, Lun ATL, Baldoni PL, Smyth GK. 2025. edgeR v4: powerful differential analysis of sequencing data with expanded functionality and improved support for small counts and larger datasets. Nucleic Acids Res 53:gkaf018. doi:10.1093/nar/gkaf018 PMC11754124 39844453

[27] Ritchie ME, Phipson B, Wu D, Hu Y, Law CW, Shi W, Smyth GK. 2015. Limma powers differential expression analyses for RNA-sequencing and microarray studies. Nucleic Acids Res 43:e47. doi:10.1093/nar/gkv007 PMC4402510 25605792

[28] Wu T, Hu E, Xu S, Chen M, Guo P, Dai Z, Feng T, Zhou L, Tang W, Zhan L, Fu X, Liu S, Bo X, Yu G. 2021. clusterProfiler 4.0: a universal enrichment tool for interpreting omics data. Innovation (Camb) 2:100141. doi:10.1016/j.xinn.2021.100141 PMC8454663 34557778

[29] Dennis G Jr, Sherman BT, Hosack DA, Yang J, Gao W, Lane HC, Lempicki RA. 2003. DAVID: database for annotation, visualization, and integrated discovery. Genome Biol 4:P3. doi:10.1186/gb-2003-4-9-r60 PMC193660 12734009

[30] Nandi M, Sikri K, Chaudhary N, Mande SC, Sharma RD, Tyagi JS. 2019. Multiple transcription factors co-regulate the *Mycobacterium tuberculosis* adaptation response to vitamin C. BMC Genomics 20:887. doi:10.1186/s12864-019-6190-3 PMC6868718 31752669

[31] Wickham H. 2016. ggplot2: elegant graphics for data analysis. Springer international publishing, Cham.

[32] Shannon P, Markiel A, Ozier O, Baliga NS, Wang JT, Ramage D, Amin N, Schwikowski B, Ideker T. 2003. Cytoscape: a software environment for integrated models of biomolecular interaction networks. Genome Res 13:2498–2504. doi:10.1101/gr.1239303 PMC403769 14597658

