# Supplementary material for: Vancomycin rescues sensitivity to isoniazid in clinical isolates of drug-resistant Mycobacterium tuberculosis
Source: Microbiol Spectr. 2026 Sep 10;14(10):e00173-26. doi: 10.1128/spectrum.00173-26 (PMC13689219; doi:10.1128/spectrum.00173-26)
Supplement: Supplementary file 1 [file spectrum.00173-26-s0001.pdf]

**Supplementary Figure 1.** Multiple sequence alignment of *ndh* (residue positions 80-300) from *Mtb* H37Rv and 14 clinical isolates presented in this study.

*[‘F’ refers to the forward strand and ‘R’ refers to the reverse strand from the same strain. Commonly mutated positions, T110 and R268, are conserved in this set of strains]*

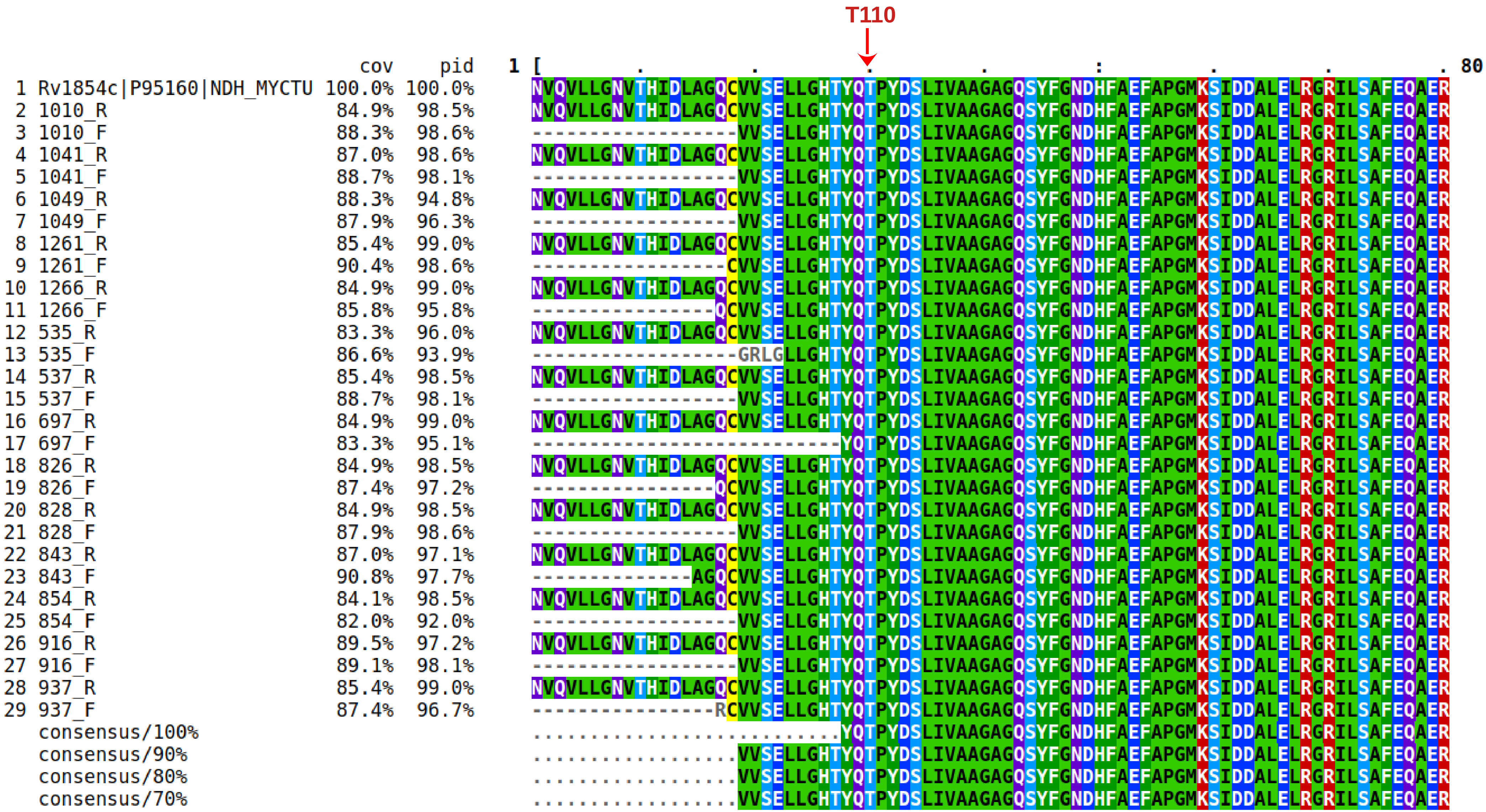

| | | cov | pid | 81 . 1 . . . . : . 160 |
|---|---|---|---|---|
| 1 | Rv1854c\|P95160\|NDH_MYCTU | 100.0% | 100.0% | SSDPERRAKLLTFTVVGAGPTGVEMAGQIAELAEHTLKGAFRHIDSTKAR-VILLDAAPAVLPPMGAKLGQRAAARLQKL |
| 2 | 1010_R | 84.9% | 98.5% | SSDPERRAKLLTFTVVGAGPTGVEMAGQIAELAEHTLKGAFRHIDSTKAR-VILLDAAPAVLPPMGAKLGQRAAARLQKL |
| 3 | 1010_F | 88.3% | 98.6% | SSDPERRAKLLTFTVVGAGPTGVEMAGQIAELAEHTLKGAFRHIDSTKAAPVILLDATPAVLPPMGAKLGQRAAARLQKL |
| 4 | 1041_R | 87.0% | 98.6% | SSDPERRAKLLTFTVVGAGPTGVEMAGQIAELAEHTLKGAFRHIDSTKAR-VILLDAAPAVLPPMGAKLGQRAAARLQKL |
| 5 | 1041_F | 88.7% | 98.1% | SSDPERRAKLLTFTVVGAGPTGVEMAGQIAELAEHTLKGAFRHIDSTK---VILLDAAPAVLPPMGAKLGQRAAARLQKL |
| 6 | 1049_R | 88.3% | 94.8% | SSDPERRAKLLTFTVVGAGPTGVEMAGQIAELAEHTLKGAFRHIDSTKAR-VILLDAAPAVLPPMGAKLGQRAAARLQKL |
| 7 | 1049_F | 87.9% | 96.3% | SSDPERRAKLLTFTVVGAGPTGVEMAGQIAELAEHTLKGAFRHIDSTK------LDATPAVLPPMGAKLGQRAAARLQKL |
| 8 | 1261_R | 85.4% | 99.0% | SSDPERRAKLLTFTVVGAGPTGVEMAGQIAELAEHTLKGAFRHIDSTKAR-VILLDAAPAVLPPMGAKLGQRAAARLQKL |
| 9 | 1261_F | 90.4% | 98.6% | SSDPERRAKLLTFTVVGAGPTGVEMAGQIAELAEHTLKGAFRHIDSTKAR-VILLDATPAVLPPMGAKLGQRAAARLQKL |
| 10 | 1266_R | 84.9% | 99.0% | SSDPERRAKLLTFTVVGAGPTGVEMAGQIAELAEHTLKGAFRHIDSTKAR-VILLDAAPAVLPPMGAKLGQRAAARLQKL |
| 11 | 1266_F | 85.8% | 95.8% | SSDPERRAKLLTFTVVGAGPTGVEMAGQIAELAEHTLKGAFRHIDSTK---------TPAVLPPMGAKLGQRAAARLQKL |
| 12 | 535_R | 83.3% | 96.0% | SSDPERRAKLLTFTVVGAGPTGVEM--QIAELAEHTLKGAFRHIDSTKAR-VILLDAAPAVLPPMGAKLGQRAAARLQKL |
| 13 | 535_F | 86.6% | 93.9% | SSDPERRAKLLTFTVVGAGPTGVEMAGQIAELAEHTLKGAFRHIDSTKDE------AAAAVLPPMGAKLGHRAAARLQKL |
| 14 | 537_R | 85.4% | 98.5% | SSDPERRAKLLTFTVVGAGPTGVEMAGQIAELAEHTLKGAFRHIDSTKAR-VILLDAAPAVLPPMGAKLGQRAAARLQKL |
| 15 | 537_F | 88.7% | 98.1% | SSDPERRAKLLTFTVVGAGPTGVEMAGQIAELAEHTLKGAFRHIDSTK---VILLDAAPAVLPPMGAKLGQRAAARLQKL |
| 16 | 697_R | 84.9% | 99.0% | SSDPERRAKLLTFTVVGAGPTGVEMAGQIAELAEHTLKGAFRHIDSTKAR-VILLDAAPAVLPPMGAKLGQRAAARLQKL |
| 17 | 697_F | 83.3% | 95.1% | SSDPERRAKLLTFTVVGAGPTGVEMAGQIAELAEHTLKGAFRHIDSTKR-------ATPAVLPPMGAKLGQRAAARLQKL |
| 18 | 826_R | 84.9% | 98.5% | SSDPERRAKLLTFTVVGAGPTGVEMAGQIAELAEHTLKGAFRHIDSTKAR-VILLDAAPAVLPPMGAKLGQRAAARLQKL |
| 19 | 826_F | 87.4% | 97.2% | SSDPERRAKLLTFTVVGAGPTGVEMAGQIAELAEHTLKGAFRHIDSTK------LDATPAVLPPMGAKLGQRAAARLQKL |
| 20 | 828_R | 84.9% | 98.5% | SSDPERRAKLLTFTVVGAGPTGVEMAGQIAELAEHTLKGAFRHIDSTKAR-VILLDAAPAVLPPMGAKLGQRAAARLQKL |
| 21 | 828_F | 87.9% | 98.6% | SSDPERRAKLLTFTVVGAGPTGVEMAGQIAELAEHTLKGAFRHIDSTK---VILLDATPAVLPPMGAKLGQRAAARLQKL |
| 22 | 843_R | 87.0% | 97.1% | SSDPERRAKLLTFTVVGAGPTGVEMAGQIAELAEHTLKGAFRHIDSTKAR-VILLDAAPAVLPPMGAKLGQRAAARLQKL |
| 23 | 843_F | 90.8% | 97.7% | SSDPERRAKLLTFTVVGAGPTGVEMAGQIAELAEHTLKGAFRHIDSTK---VILLDAAPAVLPPMGAKLGQRAAARLQKL |
| 24 | 854_R | 84.1% | 98.5% | SSDPERRAKLLTFTVVGAGPTGVEMAGQIAELAEHTLKGAFRHIDSTKAR-VILLDAAPAVLPPMGAKLGQRAAARLQKL |
| 25 | 854_F | 82.0% | 92.0% | SSDPERRAKLLTFTVVGAGPTGVEMAGQIAELAEHTLKGAFRHIDSTK-----------------GAKLGHRAAARLQKL |
| 26 | 916_R | 89.5% | 97.2% | SSDPERRAKLLTFTVVGAGPTGVEMAGQIAELAEHTLKGAFRHIDSTKAR-VILLDAAPAVLPPMGAKLGQRAAARLQKL |
| 27 | 916_F | 89.1% | 98.1% | SSDPERRAKLLTFTVVGAGPTGVEMAGQIAELAEHTLKGAFRHIDSTK---VILLDAAPAVLPPMGAKLGQRAAARLQKL |
| 28 | 937_R | 85.4% | 99.0% | SSDPERRAKLLTFTVVGAGPTGVEMAGQIAELAEHTLKGAFRHIDSTKAR-VILLDAAPAVLPPMGAKLGQRAAARLQKL |
| 29 | 937_F | 87.4% | 96.7% | SSDPERRAKLLTFTVVGAGPTGVEMAGQIAELAEHTLKGAFRHIDSTK------LDATPAVLPPMGAKLGQRAAARLQKL |
| | consensus/100% | | | SSDPERRAKLLTFTVVGAGPTGVEM..QIAELAEHTLKGAFRHIDSTK.................GAKLGpRAAARLQKL |
| | consensus/90% | | | SSDPERRAKLLTFTVVGAGPTGVEMAGQIAELAEHTLKGAFRHIDSTK........AsPAVLPPMGAKLGQRAAARLQKL |
| | consensus/80% | | | SSDPERRAKLLTFTVVGAGPTGVEMAGQIAELAEHTLKGAFRHIDSTK......LDAsPAVLPPMGAKLGQRAAARLQKL |
| | consensus/70% | | | SSDPERRAKLLTFTVVGAGPTGVEMAGQIAELAEHTLKGAFRHIDSTK...VILLDAsPAVLPPMGAKLGQRAAARLQKL |

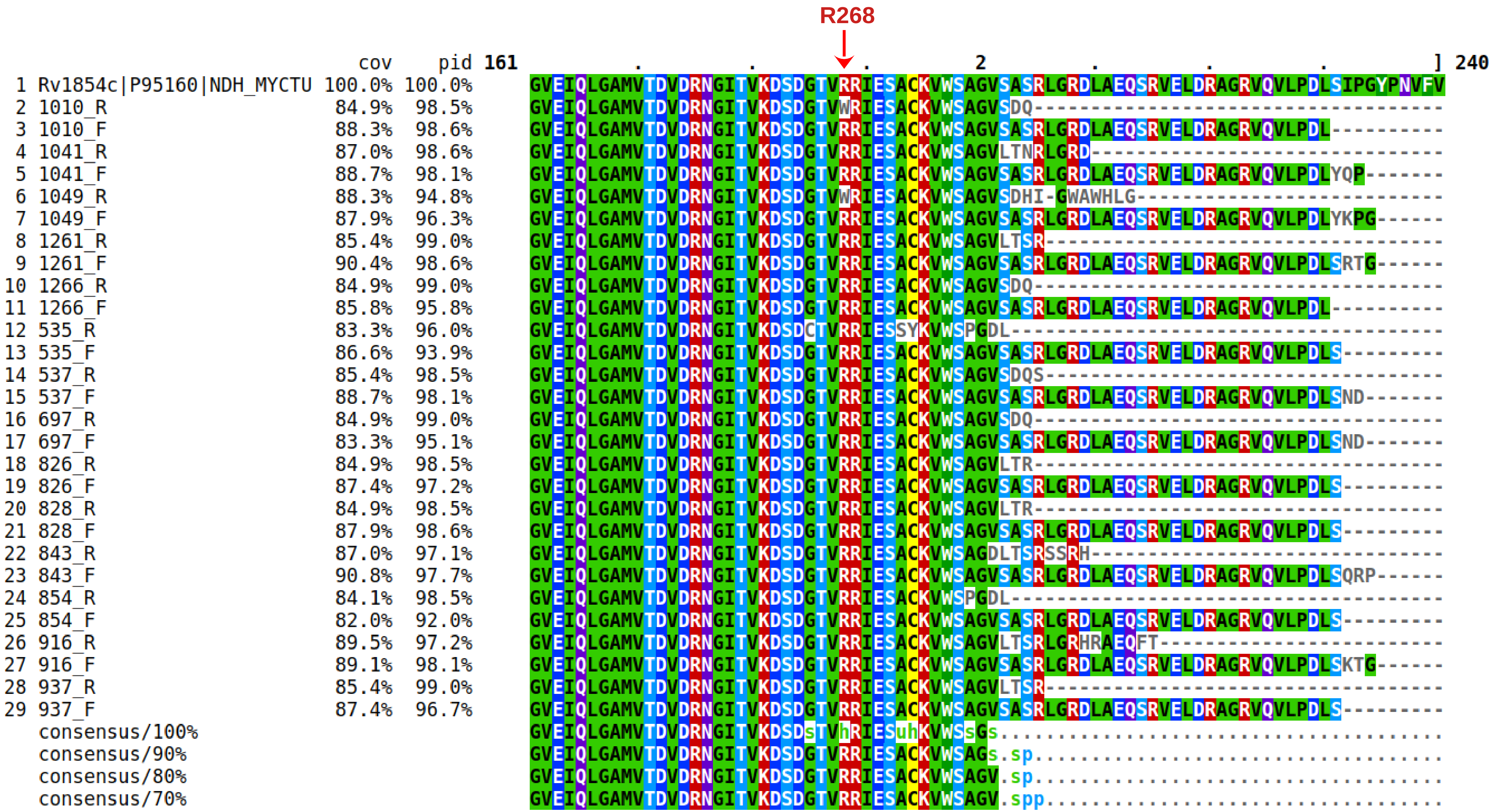

R268
cov pid 161 . . . 2 . . . ] 240
1 Rv1854c|P95160|NDH_MYCTU 100.0% 100.0% GVEIQLGAMVTDVDRNGITVKDSDGTVRRIESACKVWSAGVSASRLGRDLAEQSRVELDRAGRVQVLPDLSIPGYPNVFV
2 1010_R 84.9% 98.5% GVEIQLGAMVTDVDRNGITVKDSDGTVWRIESACKVWSAGVSDQ-------------------------------------
3 1010_F 88.3% 98.6% GVEIQLGAMVTDVDRNGITVKDSDGTVRRIESACKVWSAGVSASRLGRDLAEQSRVELDRAGRVQVLPDL----------
4 1041_R 87.0% 98.6% GVEIQLGAMVTDVDRNGITVKDSDGTVRRIESACKVWSAGVLTNRLGRD-------------------------------
5 1041_F 88.7% 98.1% GVEIQLGAMVTDVDRNGITVKDSDGTVRRIESACKVWSAGVSASRLGRDLAEQSRVELDRAGRVQVLPDLYQP-------
6 1049_R 88.3% 94.8% GVEIQLGAMVTDVDRNGITVKDSDGTVWRIESACKVWSAGVSDHI-GWAWHLG---------------------------
7 1049_F 87.9% 96.3% GVEIQLGAMVTDVDRNGITVKDSDGTVRRIESACKVWSAGVSASRLGRDLAEQSRVELDRAGRVQVLPDLYKPG------
8 1261_R 85.4% 99.0% GVEIQLGAMVTDVDRNGITVKDSDGTVRRIESACKVWSAGVLTSR-----------------------------------
9 1261_F 90.4% 98.6% GVEIQLGAMVTDVDRNGITVKDSDGTVRRIESACKVWSAGVSASRLGRDLAEQSRVELDRAGRVQVLPDLSRTG------
10 1266_R 84.9% 99.0% GVEIQLGAMVTDVDRNGITVKDSDGTVRRIESACKVWSAGVSDQ-------------------------------------
11 1266_F 85.8% 95.8% GVEIQLGAMVTDVDRNGITVKDSDGTVRRIESACKVWSAGVSASRLGRDLAEQSRVELDRAGRVQVLPDL----------
12 535_R 83.3% 96.0% GVEIQLGAMVTDVDRNGITVKDSDCTVRRIESSYKVWSPGDL---------------------------------------
13 535_F 86.6% 93.9% GVEIQLGAMVTDVDRNGITVKDSDGTVRRIESACKVWSAGVSASRLGRDLAEQSRVELDRAGRVQVLPDLS---------
14 537_R 85.4% 98.5% GVEIQLGAMVTDVDRNGITVKDSDGTVRRIESACKVWSAGVSDQS------------------------------------
15 537_F 88.7% 98.1% GVEIQLGAMVTDVDRNGITVKDSDGTVRRIESACKVWSAGVSASRLGRDLAEQSRVELDRAGRVQVLPDLSND-------
16 697_R 84.9% 99.0% GVEIQLGAMVTDVDRNGITVKDSDGTVRRIESACKVWSAGVSDQ-------------------------------------
17 697_F 83.3% 95.1% GVEIQLGAMVTDVDRNGITVKDSDGTVRRIESACKVWSAGVSASRLGRDLAEQSRVELDRAGRVQVLPDLSND-------
18 826_R 84.9% 98.5% GVEIQLGAMVTDVDRNGITVKDSDGTVRRIESACKVWSAGVLTR-------------------------------------
19 826_F 87.4% 97.2% GVEIQLGAMVTDVDRNGITVKDSDGTVRRIESACKVWSAGVSASRLGRDLAEQSRVELDRAGRVQVLPDLS---------
20 828_R 84.9% 98.5% GVEIQLGAMVTDVDRNGITVKDSDGTVRRIESACKVWSAGVLTR-------------------------------------
21 828_F 87.9% 98.6% GVEIQLGAMVTDVDRNGITVKDSDGTVRRIESACKVWSAGVSASRLGRDLAEQSRVELDRAGRVQVLPDLS---------
22 843_R 87.0% 97.1% GVEIQLGAMVTDVDRNGITVKDSDGTVRRIESACKVWSAGDLTSRSSRH-------------------------------
23 843_F 90.8% 97.7% GVEIQLGAMVTDVDRNGITVKDSDGTVRRIESACKVWSAGVSASRLGRDLAEQSRVELDRAGRVQVLPDLSQRP------
24 854_R 84.1% 98.5% GVEIQLGAMVTDVDRNGITVKDSDGTVRRIESACKVWSPGDL---------------------------------------
25 854_F 82.0% 92.0% GVEIQLGAMVTDVDRNGITVKDSDGTVRRIESACKVWSAGVSASRLGRDLAEQSRVELDRAGRVQVLPDLS---------
26 916_R 89.5% 97.2% GVEIQLGAMVTDVDRNGITVKDSDGTVRRIESACKVWSAGVLTSRLGRHRAEQFT-------------------------
27 916_F 89.1% 98.1% GVEIQLGAMVTDVDRNGITVKDSDGTVRRIESACKVWSAGVSASRLGRDLAEQSRVELDRAGRVQVLPDLSKTG------
28 937_R 85.4% 99.0% GVEIQLGAMVTDVDRNGITVKDSDGTVRRIESACKVWSAGVLTSR-----------------------------------
29 937_F 87.4% 96.7% GVEIQLGAMVTDVDRNGITVKDSDGTVRRIESACKVWSAGVSASRLGRDLAEQSRVELDRAGRVQVLPDLS---------
consensus/100% GVEIQLGAMVTDVDRNGITVKDSDsTVhRIESuhKVWSsGs.......................................
consensus/90% GVEIQLGAMVTDVDRNGITVKDSDGTVRRIESACKVWSAGs.sp....................................
consensus/80% GVEIQLGAMVTDVDRNGITVKDSDGTVRRIESACKVWSAGV.sp....................................
consensus/70% GVEIQLGAMVTDVDRNGITVKDSDGTVRRIESACKVWSAGV.spp...................................
